# In-patient rehabilitation: clinical outcomes and cost implications

**DOI:** 10.1192/pb.bp.114.049858

**Published:** 2016-02

**Authors:** Mel Bunyan, Yogesh Ganeshalingam, Ehab Morgan, Donvé Thompson-Boy, Rebekah Wigton, Frank Holloway, Derek K. Tracy

**Affiliations:** 1 Oxleas NHS Foundation Trust, London, UK; 2 King's College London; 3 South London and Maudsley NHS Foundation Trust

## Abstract

**Aims and method** A retrospective evaluation was undertaken of the clinical and economic effectiveness of three in-patient rehabilitation units across one London National Health Service trust. Information on admission days and costs 2 years before and 2 years after the rehabilitation placement, length of rehabilitation placement and the discharge pathway was collected on 22 service users.

**Results** There were statistically significant reductions in hospital admission days in the 2 years following rehabilitation compared with the 2 years before, further reflected in significantly lower bed costs. Longer length of rehabilitation placement was correlated with fewer admission days after the placement. A substantial proportion of the sample went into more independent living, some with no further admissions at follow-up.

**Clinical implications** The findings suggest that in-patient rehabilitation is both clinically and cost effective: if benefits are sustained they will offset the cost of the rehabilitation placement.

Mental health rehabilitation services work with ‘low volume, high need’ individuals^1^ with complex long-term mental health and social care needs. National policy on appropriate levels of in-patient rehabilitation provision has been lacking,^2,3^ although the majority of mental health trusts provide such care.^4^ The National Service Framework for Mental Health^5^ focused on specialist community services, and a relative reduction of rehabilitation services has followed its publication.^2,6,7^

Mental health services in the UK are facing the uncertain challenges of moving to clinical commissioning groups, with the parallel drive to evidence outcomes (or at least activity) through payment by results. This must be further filtered through the reality of a mandated £20 billion in efficiency savings in the National Health Service (NHS) by 2014,^8^ creating competition for ever-reduced resources.^9^ There are specific dangers for rehabilitation services in this context: a lack of clear governmental policy support and a limited – though generally positive – scientific evidence base could result in a loss of money to competing services. Commissioners' knowledge of the role and effectiveness of rehabilitation is uncertain, although specific guidance to assist commissioning has been produced.^1^

In this study, we explored objective clinical (bed use), economic (bed costs) and psychosocial functioning outcome data in three in-patient rehabilitation units across a single NHS mental health trust. We hypothesised that there would be measurable improvements in individuals' lives provided in a cost-effective manner, and that data could help inform commissioning decisions about rehabilitation service provision.

## Method

The study retrospectively explored clinical and costing markers in 22 individuals sequentially discharged from three in-patient rehabilitation units in a single NHS trust in the 2 years prior, the time during and the 2 years after rehabilitation care.

### Rehabilitation units

Within a wider rehabilitation service, Oxleas NHS Foundation Trust has three in-patient rehabilitation units, one in each of three London boroughs that encompass both inner and outer London (covering a total population of 796 000),^10^ providing a total of 46 placements. They each provide 24-hour nursing care, offer a range of professional inputs and can accommodate patients detained under the Mental Health Act 1983.

### Participants

The sample was drawn from the 24 individuals who were discharged from the three units between 1 October 2009 and 30 September 2010: two patients were excluded as they had spent fewer than 6 weeks on a unit, leaving a sample of 22. The mean length of admission was 701 days (s.d. = 385, range 132-1434). Referrals came from acute psychiatric wards (*n* = 14), closed rehabilitation units (*n* = 2), forensic in-patient units (*n* = 2) and a private hospital (*n* = 1). Three people were referred directly from the community, although these were more ‘intensive’ community services rather than standard community mental health teams. Mean age was 49 years (s.d. = 12.23, range 22–71). All had a primary psychotic diagnosis, with paranoid schizophrenia most common (*n* = 13); 12 had at least one secondary diagnosis and these included substance misuse (*n* = 4), intellectual disability (*n* = 4), personality disorder (*n* = 3) and an anxiety disorder (*n* = 2). Twelve patients were detained under Section 3 of the Mental Health Act.

### Procedure

Ethics approval for retrospective data collection and dissemination was obtained through the trust research and development office. Data were collected on: bed occupancy and costs (amalgamated costs are shown for acute, psychiatric intensive care unit, low secure, private and rehabilitation beds), risk and meaningful social activities in the 2 years before and 2 years after the placement, length of stay in the rehabilitation unit, and the pathway from referral to discharge. Information was obtained from the trust's electronic record system, RiO: where information prior to RiO's implementation in 2006 was needed, this was taken from paper notes. Differences in bed occupancy and costs were assessed for significance using paired *t*-tests and Spearman's *r_s_* correlation coefficient; α = 0.05 was adopted for all comparisons.

## Results

### Admission and discharge data with costs

**Fig. 1:**
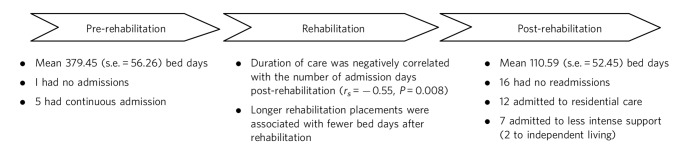
Mean number of admission days in the 2 years pre- and 2 years post-rehabilitation.

Patients demonstrated a statistically significant reduction in admission days after rehabilitation, spending significantly less time in hospital in the 2 years after rehabilitation than the 2 years before (*t*_(21)_ = 3.052, *P* = 0.006) (Fig. 1).

Fitting with a reduction in admission days, the costs post-rehabilitation were statistically significantly lower than the costs of admissions pre-rehabilitation (Table 1). Linear regression analysis incorporating both age and gender as predictors failed to identify either factor as a significant predictor of bed days or costs.

**Table 1:** Admission costs per year pre- and post-rehabilitation

	Pre-rehabilitation	Post-rehabilitation	Statistics
Individual cost, mean (s.e.)	£66 000 (£10 000)	£18 000 (£9000)	*t*_(21)_ = 3.200, *P* = 0.004

Total cost (*n* = 22)	£1324 000	£386 000	

The five individuals who had been in continuous admission for the 2 years before rehabilitation remained in rehabilitation care longer than average (mean admission 953 *v.* 701 days). Of this subgroup four were discharged to residential care, one to an independent flat, and three had no admissions in the follow-up period.

Five individuals required 3–4 years of rehabilitation input, and this subgroup continued to require a substantial amount of post-discharge care, with four discharged into residential care, although none were readmitted in the 2-year follow-up period. Of the 11 who stayed less than 18 months, 2 were discharged into long-term in-patient care (1 forensic team, 1 older adults' service): these were the only 2 patients in the sample who went into continuous in-patient care and, speculatively, the shorter duration of input may represent a clinical recognition that the rehabilitation programme was not going to progress effectively.

### Psychosocial functioning

Pre-rehabilitation there was no information available in the notes for three people regarding their functioning, and for ten others, despite detailed information being available, there was no evidence of engagement in regular daytime activities. Of the remainder, where there was evidence of regular activities: three were having regular contact with family members, five were attending ward groups or occupational therapy activities and one was doing voluntary work. Greater levels of activity were recorded in the period post-rehabilitation, with only three patients having no evidence of any form of meaningful activity. Ten were engaged in community groups, three were service user representatives, two were engaged with ward occupational therapy, two had re-established meaningful links with their families, one was at college, and one was undertaking voluntary work. Quite a few were doing more than one activity; for the purposes of this report, we documented what we deemed to be each person's highest level of achievement.

### Risk

Most of the sample had complex risk histories: eight had a forensic history including firesetting, assaults, actual bodily harm, carrying weapons and shoplifting; five had a history of substance misuse; nine had a history of substantial acts of self-harm or suicide attempts; and almost all had some degree of self-neglect. Sixteen of the 22 individuals had one or two, and 3 individuals had three to five major risk incidents (defined as self-neglect, self-harm, damaging property, verbal or physical abuse, absconding or inappropriate sexual behaviour) in the 2 years preceding their rehabilitation placement. Post-rehabilitation half the sample had no major risk incidents in the follow-up period, five had one major risk incident, four had two such incidents, and two individuals had four major risk events.

## Discussion

A limited evidence base exists for in-patient rehabilitation services. About 80% of rehabilitation services users have a primary diagnosis of psychosis,^11^ and the rationale for input may include treatment resistance, comorbidities such as neurodevelopmental disorders and substance misuse, behavioural disturbances, and an inability to effect discharge from an acute ward.^12^ It has traditionally been argued that the majority of rehabilitation patients are not hard to engage despite their needs^7^ – unlike a typical assertive outreach cohort – and under current UK NHS criteria will usually fall under payment by results ‘cluster 13’. However, this assertion might be challenged given the general reduction in in-patient beds and often in the provision of assertive outreach services, and rehabilitation case-loads might be changing with time. The first national survey of 133 rehabilitation units in England^11^ found a mean of 14 beds per unit and 16 admissions in the previous year. Median service user characteristics included an age of 40, an admission of 18 months' duration, a 13-year history of contact with mental health services and 4 previous in-patient admissions.

A 5-year prospective follow-up of 72 individuals ‘difficult to place’ following a hospital closure^13^ demonstrated positive outcomes of greater independence, fewer episodes of aggression and problematic behaviour from a rehabilitative approach. Participants showed significant gains in skills despite persisting or worsening symptoms but a ‘slow-stream’ approach was required, with improvements taking over a year to manifest. Killaspy & Zis^14^ analysed clinical outcome data on 141 users of an inner London rehabilitation service retrospectively: 40% had positive outcomes 5 years after initial assessment – defined as achieving and sustaining a less supported placement – with 10% moving to independent accommodation and sustaining a tenancy, and 27% had unchanged support. Adherence to medication was the most significant factor positively affecting outcomes at 5 years, although, conflicting with the finding of Bredski *et al*,^15^ a longer time from first contact with mental health services to contact with rehabilitation services was also associated with better outcomes. In the national study of rehabilitation units in England^11^ positive scores in the seven domains measured by the Quality Indicator for Rehabilitative Care (QuIRC)^16^ were associated with better subjective experiences of care and the therapeutic environment.

A case–control study (34 discharged, 31 non-discharged residents) by Bredski *et al*^15^ demonstrated that serious self-harm, high-dose and polypharmacy antipsychotic use, past forensic input and longer past duration of hospital admission(s) were significantly associated with non-discharge. Killaspy *et al*^11^ showed that older patient age was significantly associated with worse outcomes. This national survey identified delayed discharges in 14% of rehabilitation patients, whereas a lack of suitable move-on accommodation – particularly from lower intensity community units – was noted in another, ten-unit survey by Cowan *et al*:^17^ over half of the patients had no clear community team follow-up. A lack of appropriate follow-up for those with high levels of disability, a quarter of whom ended up under the care of assertive outreach teams, was identified as a gap.

Our data indicate that the majority of people using the three evaluated rehabilitation units appeared to derive significant benefit in terms of increased stability, with fewer hospital admissions and the ability to live in more independent settings. The duration of rehabilitation placement varied, tailored to individual need rather than specific time scales, with a considerable range in duration, although the majority moved through the units in well under 2 years. Interestingly, a greater duration of rehabilitation input was significantly associated with better outcomes as measured by readmission data. Although on one level this might appear intuitive as a proxy marker of quanta of clinical input, it is also reasonable to consider that those least well and most in need would necessitate the greatest input and potentially have worse outcomes – though as noted, two individuals who were transferred to continuous in-patient care had such a move made relatively early. These data thus fit with Trieman & Leff's^13^ concept that rehabilitation care takes time to effect change. It was particularly pleasing to see that a number of people who had spent extended periods of their lives in hospital were able to move into the community and maintain this progress in the follow-up period, and that 16 of the sample had no admissions during this time. From a service perspective, this led to cost savings on hospital admissions of around £48 000 a year per individual treated in the follow-up period, compared with the 2 years pre-rehabilitation. This of course must be weighed against the cost of the rehabilitation placement: in these specific units this would be recouped within 3.5 years of discharge if such gains were sustained, although we do not yet have such longer-term clinical data.

It was harder to determine the secondary measures of whether people had been able to pursue other personally meaningful goals and aspirations, and to reliably assay their risk profile: the sample size was small and validated scales were not utilised. Overall, the findings are concordant with the somewhat similarly designed retrospective work by Killaspy & Zis^14^ in terms of positive outcomes following rehabilitation care, although that larger study focused more on the nature of placement after rehabilitation rather than admission days and costs, and it was powered to evaluate factors correlated with outcome such as medication adherence.

### Limitations

A major limitation of this work is the lack of a control group: although the data show reduced readmissions and related cost savings, as well as improvements in psychosocial functioning and reduction in risk, it remains possible that such gains would have occurred without the rehabilitation-specific input. A randomised control trial would be necessary to determine causality. Hospital admission and cost are clearly directly related factors, although we have reported them separately, and the former is only an indirect marker of clinical state. However, particularly in the current economic climate, much service-related research has neglected the issues of costings, and we believe our model could be applied to parallel services such as assertive outreach, early intervention and crisis teams, to assess their cost-effectiveness over time. Nevertheless, the opportunity cost of standard community ‘treatment as usual’ is not factored into the overall costs in this current work.

Data collection was retrospective and analysis of psychosocial functioning and risk was descriptive rather than through the use of objective and validated scales. Linear regression analysis did not identify age or gender as predictors of bed days or costs, but the sample size was too small to allow any meaningful attempt to evaluate clinical or demographic predictors of outcome and caution should be exercised in the interpretation of this negative finding. However, this was not a primary study aim, and we note the general lack of such markers both in mental health rehabilitation and more general psychosis studies to date. Finally, although this study covered three boroughs, encompassing both inner and outer London, we recognise this is from a single NHS trust in one city, which may limit the generalisability of the results.

### Rehabilitation services – future outlook

Rehabilitation services evolved as a development of the broader process of deinstitutionalisation and the end of the asylum era,^18,19^ but have suffered in recent years in relation to specialist community teams. Assertive outreach, early intervention and crisis teams are themselves open to challenge about their evidence base and effectiveness,^20^ but they appear in favour with service developers and national drivers for change, and might be perceived as producing more active research. Rehabilitation might be relatively ‘unfashionable’,^3^ but if this is the case then the rehabilitation psychiatry subspecialty must accept some responsibility for this. Rehabilitation has historically been an under-researched branch of mental health,^4,17^ despite the cost of such services^21^ and the obvious challenge of severe and enduring mental distress.^22,23^ Killaspy *et al*'s^4^ description of the field as an ‘evidence free zone’ remains, if no longer wholly accurate, nevertheless still apposite a decade after the statement was made.

In the face of clinical commissioning groups and payment by results, continuing practice as usual is not a valid proposition for any branch of mental health, and recent history augurs badly for rehabilitation without active, ongoing self-evaluation to justify continuance – let alone increase – of funding. However, there are positives such as the development of the QuIRC toolkit^16^ and the large multi-site Rehabilitation Effectiveness for Activities for Life (REAL) study in progress.^24^ Some of the difficulties in identifying demographic or clinical predictors of outcome are common to the larger field of psychosis research,^25,26^ but the evidence that does exist is broadly favourable in terms of rehabilitation input producing positive outcomes.

Psychosis research presents new developments in many areas, from new pharmacotherapeutics,^27-29^ neuroimaging^30,31^ and nascent genetics work,^32^ to positive results for individual cognitive–behavioural therapy^33^ and family systems approaches,^34^ sometimes without psychotropic medication. However, excitement about these assessment and therapeutic tools can be juxtaposed with the reality that despite advances in treatments and knowledge, psychosocial disability remains as much a problem now as it did half a century ago, and many diagnosed with psychosis continue to struggle to live satisfying and meaningful lives.^35^ Although learning to place the lived experience of the patient at the heart of our thinking is perhaps a more recent concept, the recovery philosophy has become a guiding principle for mental health services.^36,37^ Of all fields of mental health practice, the rehabilitation tradition may have the longest convergence with the recovery model, supporting people who face the greatest mental health challenges in establishing meaningful lives.^38^ We believe our data support the evidence for rehabilitation services, which continue to be neglected in policy and local planning. Rehabilitation embodies the humanistic heart of mental health: it has, perhaps, just been too silent for too long. The arrival of clinical commissioning groups and payment by results could present an opportunity to help change this.
